# 3.0T MR扩散加权成像对肺实性良恶性病变的鉴别诊断效能及b值优化探讨

**DOI:** 10.3779/j.issn.1009-3419.2011.11.04

**Published:** 2011-11-20

**Authors:** 伟栋 李, 东 李, 海东 刘, 荣超 周, 长超 冯, 艳 马, 铁链 于

**Affiliations:** 1 300052 天津，天津医科大学总医院放射科 Department of Radiology, Tianjin Medical University General Hospital, Tianjin 300052, China; 2 300100 天津，天津市中心妇产科医院放射科 Department of Radiology, Tianjin Central Hospital of Gynecology and Obstetrics, Tianjin 300100, China; 3 060002 秦皇岛，秦皇岛市第一医院放射科 Department of Radiology, the First Hospital of Qinhuangdao City, Qinhuangdao 060002, China

**Keywords:** 肺, 扩散加权成像, 磁共振成像, 表观扩散系数, Lung, Diffusion-weighted imaging, Magnetic resonance imaging, Apparent diffusion coefficient

## Abstract

**背景与目的:**

磁共振扩散加权成像（diffusion-weighted imaging, DWI）是唯一能在活体检测组织内水分子扩散运动的无创影像检查技术，能在宏观成像中反映活体组织中水分子的微观扩散运动。本研究旨在探讨3.0T磁共振成像（magnetic resonance imaging, MRI）DWI联合相控阵线圈和并行阵列采集空间敏感度编码技术（array spatial sensitivity encoding technique, ASSET）对肺实性良恶性病变的鉴别诊断效能，并优化最佳b值。

**方法:**

经病理或临床随访证实的20例肺良性病变和96例肺恶性肿瘤（共120个病灶）在3.0T MR扫描仪上行T2加权像（T2 weighted imaging, T2WI）、T1加权像（T1 weighted imaging, T1WI）、脂肪抑制T2WI以及不同b值DWI（200 s/mm^2^、500 s/mm^2^、800 s/mm^2^、1, 000 s/mm^2^）扫描，得到各b值的DWI图和表观扩散系数（apparent diffusion coefficient, ADC）图，分别测量各b值下病变的DWI信号强度、ADC值，比较各b值组的信噪比（signal-to-noise ratio, SNR）、对比噪声比（contrast-to-noise ratio, CNR）、ADC值，并绘制各b值的受试者操作特征曲线（receiver operating characteristic curve, ROC），得出ADC值对肺实性良恶性病变的鉴别诊断效能，优化DWI诊断肺部实性良恶性病变的最佳b值。

**结果:**

不同b值组间SNR、CNR差异均有统计学意义（*P* < 0.001, *P*=0.002）。肺良性和恶性病变组ADC值均随b值增加而逐渐变小，差异有统计学意义（*P* < 0.001, *P* < 0.001）。4组不同b值的ROC曲线下面积（area under curve, AUC）分别为0.831、0.876、0.813、0.785，均有诊断意义（AUC > 0.5）；b=500 s/mm^2^时获得的ADC值的诊断效能最大，鉴别良恶性病变的最佳阈值为1.473×10^-3^ mm^2^/s，敏感度和特异度分别为80%和84%。

**结论:**

3.0T MR DWI联合相控阵线圈和ASSET技术对肺实性良恶性病变的鉴别诊断有较高价值，b=500 s/mm^2^时获得的ADC值诊断效能较高。

## Abstract (en)

**Background and objective:**

Diffusion is caused by random translational molecular motion, also known as Brownian water motion. Diffusion-weighted imaging (DWI) is the only imaging method that can be used to evaluate the diffusion process *in vivo*. The aim of this study is to evaluate 3.0T magnetic resonance imaging (MRI) DWI with phased-array coil and the array spatial sensitivity encoding technique (ASSET) of diagnosis potency in the discrimination of pulmonary solid benign lesions and malignant tumors. This study also aims to optimize b value.

**Methods:**

One hundred and sixteen patients with 120 lesions confirmed by pathology and clinical diagnosis underwent T2 weighted imaging (T2WI), T1 weighted imaging, T2WI fat suppression, and DWI (diffusion factors of 200 s/mm^2^, 500 s/mm^2^, 800 s/mm^2^, 1, 000 s/mm^2^) examinations by ASSET with 3.0T MR. The signal intensity of DWI images and the apparent diffusion coefficient (ADC) values of the lesions were measured. Signal-to-noise ratio (SNR), contrast-to-noise ratio (CNR), and ADC were compared among different b values. Receiver operating characteristic (ROC) curves were analyzed and the b values were optimized.

**Results:**

Both the SNR and CNR significantly differed among varied b values (*P* < 0.001, *P*=0.002). The ADC values of pulmonary solid benign lesions and malignant tumors were gradually reduced with increasing b value, and the differences were statistically significant (*P* < 0.001, *P* < 0.001). ROC analysis shows that the area under curve (AUC) values were 0.831, 0.876, 0.813, 0.785 (b=200 s/mm^2^, 500 s/mm^2^, 800 s/mm^2^, 1, 000 s/mm^2^, respectively). The AUC with a b value of 500 s/mm^2^ was the largest. The optimal threshold of ADC was 1.473×10^-3^ mm^2^/s, and the sensitivity and specificity were 80% and 84%, respectively.

**Conclusion:**

3.0T MR DWI with phased-array coil and ASSET has moderate diagnosis potency in differentiating pulmonary solid benign lesions and malignant tumors. The optimal b value is 500 s/mm^2^.

磁共振扩散加权成像（diffusion-weighted imaging, DWI）是目前唯一能够在体检测水分子微观运动的无创性功能成像技术，在头颈部^[1]^及腹部^[2]^显示出较高的临床应用价值。近年来随着磁共振软硬件设备和成像技术的快速发展，尤其是3.0T磁共振成像（magnetic resonance imaging, MRI）扫描仪的临床应用，其信噪比（signal-to-noise ratio, SNR）及分辨率提高，且扫描速度加快，为DWI在胸部的应用提供了良好基础。DWI在肺部病变，特别是肺癌的检出、诊断、分期和疗效评估等方面越来越多地受到关注^[3-6]^。本研究旨在探讨3.0T MR DWI联合相控阵线圈和并行采集阵列空间敏感度编码技术（array spatial sensitivity encoding technique, ASSET）对肺实性良恶性病变的鉴别诊断效能，并优化最佳b值。

## 1 资料与方法

### 1.1 研究对象

病例入组标准：①胸部CT检查发现肺内实性结节或肿块，且直径 > 1.5 cm; ②患者一般状况良好，能配合完成检查; ③无MR检查禁忌症。全部病例均经患者同意，签署书面知情同意书。2009年6月-2011年5月在天津医科大学总医院就诊且符合上述标准的116例患者，共计120个病灶纳入研究，男性69例，女性47例，年龄36岁-85岁，平均（58.3±9.6）岁。病灶最大径线1.5 cm-12.2 cm，平均（5.5±2.5）cm。

### 1.2 MR检查

采用GE HD-X 3.0T超导型MR扫描仪和Torsopa相控阵表面线圈进行横断面扫描：①快速弛豫快速自旋回波脉冲序列（fast relaxation fast spin echo, FRFSE）T2加权成像（T2 weighted imaging）（FRFSE T2WI），呼吸触发和心电触发（R波触发），TR/TE（8, 000-8, 571）ms/（86-96）ms，层厚/间隔4.0 mm/1.0 mm，NEX 2，ETL 20，FOV 42 cm，矩阵256×160;②双反转快速自旋回波（dual inversion recovery fast spin echo）T1WI，心电触发（R波触发），TR/TE（1, 120-1, 760）ms/（4.1-6.2）ms，NEX 0.5，ETL 24，FOV 42 cm，矩阵256×160;③预饱和脂肪抑制FRFSE T2WI; ④DWI：先行ASSET校准扫描，然后采用单次激发自旋回波-回波平面成像序列（spin echo-echo planar imaging, SE-EPI）行DWI扫描，在自由呼吸状态下采集图像，b值分别取0 s/mm^2^、200 s/mm^2^、500 s/mm^2^、800 s/mm^2^、1, 000 s/mm^2^，同时在X、Y、Z轴3个方向上施加敏感梯度脉冲。

### 1.3 图像后处理及数据测量

使用AW4.3工作站的Functool 4.5.5软件包对图像进行后处理，获得肺内实性病变的DWI图、表观扩散系数（apparent diffusion coefficient, ADC）图。参考T2WI或脂肪抑制T2WI、T1WI和DWI图，选择病灶信号强度最大且最均匀的层面，通过圆形或椭圆形感兴趣区（region of interest, ROI）测量病变区的DWI信号强度（S_病变_）和ADC值。所取ROI包括病灶最大径线的60%以上，并尽可能包括最大信号强度中心区域，避开病变边缘和肉眼可辨的坏死区。同一病例各b值图像设置同样的ROI。应用同样大小ROI测量病灶同层面胸壁肌肉DWI信号强度值（S_肌肉_）、图像背景噪声（背景信号强度的标准差，SD_噪声_）。所有ROI测量均进行3次，取平均值作为最终测量值。信噪比和对比噪声比（contrast-to-noise ratio, CNR）计算公式分别为：SNR=S_病变_/SD_噪声_，CNR=（S_病变_-S_肌肉_）/SD_噪声_。

### 1.4 统计分析方法

使用SPSS 13.0统计分析软件。采用随机区组设计方差分析比较不同b值组病灶的SNR、CNR和良、恶性病变ADC值。采用受试者操作特征曲线（receiver operating characteristic curve, ROC）分析不同b值组ADC值对肺良、恶性病变的鉴别诊断效能。以*P* < 0.05为差异有统计学意义。

## 2 结果

### 2.1 良恶性病变组情况

112例病例经病理（手术、支气管镜活检或穿刺活检）证实，4例经临床资料证实（均为良性，3例抗炎后消失，1例结核菌素纯蛋白衍生物（purified protein derivative, PPD）试验强阳性并经抗结核治疗好转）。恶性病变组共96例，100个病灶，其中肺癌93例（鳞癌31例，腺癌31例，细支气管肺泡癌5例，小细胞癌19例，腺鳞癌2例，肉瘤样癌1例，肉瘤1例，低分化癌2例，大细胞癌1例），转移瘤4例，7个瘤灶（结肠癌肺转移2例，5个病灶，小腿恶性神经鞘膜瘤单发肺转移1例，肺癌伴同侧肺单发转移瘤1例，该例也计入上述肺癌病例）; 良性病变组共20例，20个病灶，其中化脓性炎性肿块4例，结核球4例，结节病4例，错构瘤2例，硬化性血管瘤2例，机化性肺炎、炎性假瘤、神经鞘瘤、神经纤维瘤各1例。

### 2.2 不同b值组DWI的SNR和CNR比较

不同b值组DWI的SNR和CNR见表 1。随着b值的增加，SNR逐渐下降（图 1，图 2），CNR则呈现出先增大后减小的趋势，b值为500 s/mm^2^时CNR最大，不同b值组间SNR、CNR差异均有统计学意义（*F*=54.457, *P* < 0.001; *F*=4.922, *P*=0.002）。两两组间比较显示b值为800 s/mm^2^与1, 000 s/mm^2^组间SNR无统计学差异，余两组之间均有统计学差异; b值为200 s/mm^2^与500 s/mm^2^、200 s/mm^2^与800 s/mm^2^、800 s/mm^2^与1, 000 s/mm^2^组间CNR无统计学差异，余两组之间均有统计学差异。

**1:** 不同b值组间SNR、CNR SNR and CNR with different b values

b values (s/mm^2^)	SNR	CNR
200	67.548±38.049	23.773±24.641
500	48.071±27.443	25.556±21.814
800	33.219±20.191	19.065±17.110
1, 000	26.777±16.799	16.916±14.929
SNR: signal-to-noise ratio; CNR: contrast-to-noise ratio. The SNR differed significantly between any two b values but between 800 s/mm^2^ and 1, 000 s/mm^2^ (*P* > 0.05); The CNR differed significantly between any two b values but between 200 s/mm^2^ and 500 s/mm^2^, 200 s/mm^2^ and 800 s/mm^2^, and 800 s/mm^2^ and 1, 000 s/mm^2^ (*P* > 0.05).		

**1:**
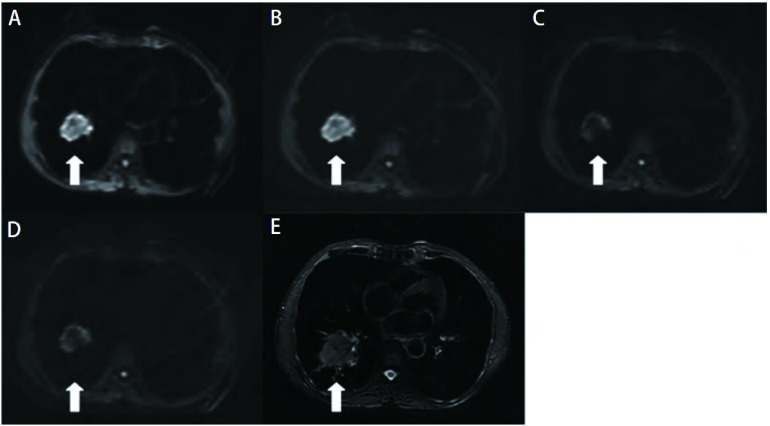
不同b值组肺内病变DWI图和T2WI脂肪抑制图。DWI：磁共振扩散加权成像; T2WI：T2加权成像。A-D：b值分别为200 s/mm^2^、500 s/mm^2^、800 s/mm^2^、1, 000 s/mm^2^时病变DWI图。随b值升高，病变SNR逐渐降低; E：T2WI脂肪抑制图，右下叶不规则肿块，呈高信号（病理诊断为低分化鳞癌）。 DWI of the pulmonary lesion with different b values and T2WI fat-suppression. DWI: diffusion-weighted imaging; T2WI: T2 weighted imaging. A-D: b values were 200 s/mm^2^ (A), 500 s/mm^2^ (B), 800 s/mm^2^ (C), and 1, 000 s/mm^2^ (D) respectively. As b value increased, the SNR of the lesion descended (arrow); E: T2WI fat-suppression showed an irregular hyperintense mass in the right inferior lobe (poorly differentiated squamous cell carcinoma histologically confirmed) (arrow).

**2:**
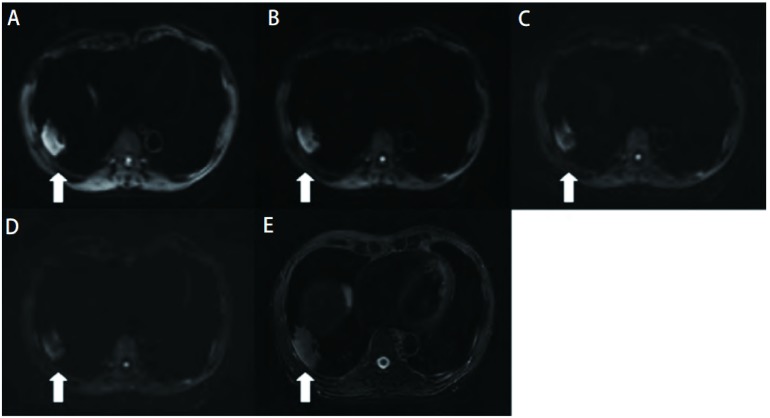
不同b值组肺内病变DWI图和T2WI脂肪抑制图。A-D：b值分别为200 s/mm^2^、500 s/mm^2^、800 s/mm^2^、1, 000 s/mm^2^时病变DWI图。随b值升高，病变SNR逐渐降低; E：T2WI脂肪抑制图，右下叶胸膜下不规则肿块，呈高信号（病理诊断为机化性肺炎）。 DWI of the pulmonary lesion with different b values and T2WI fat-suppression. A-D: b values were 200 s/mm^2^ (A), 500 s/mm^2^ (B), 800 s/mm^2^ (C), and 1, 000 s/mm^2^ (D) respectively. As b value increased, the SNR of the lesion descended (arrow); E: T2WI fat-suppression showed an irregular hyperintense mass in the right inferior lobe (organized pneumonia histologically confirmed) (arrow).

### 2.3 不同b值组病变ADC值比较

不同b值组良恶性病变ADC值比较见表 2。良性和恶性组ADC值均随b值增加逐渐变小，且差异均有统计学意义（*F*=9.389, *P* < 0.001; *F*=44.384, *P* < 0.001）。两两组间比较分别显示良性和恶性组b值为500 s/mm^2^与800 s/mm^2^、800 s/mm^2^与1, 000 s/mm^2^间ADC均无统计学差异，余两组之间均有统计学差异。

**2:** 不同b值组间良性和恶性病变ADC值（×10^-3^ mm^2^/s） ADC with different b values of benign lesions and malignant tumors (×10^-3^ mm^2^/s)

b values (s/mm^2^)	Benign lesions (*n*=20)	Malignant tumors (*n*=100)
200	2.119±0.428	1.557±0.423
500	1.816±0.425	1.236±0.272
800	1.612±0.420	1.186±0.229
1, 000	1.454±0.403	1.109±0.210
The ADC of benign lesions differed significantly between any two b values but between 500 s/mm^2^ and 800 s/mm^2^, 800 s/mm^2^ and 1, 000 s/mm^2^ (*P* > 0.05); The ADC of malignant tumors differed significantly between any two b values but between 500 s/mm^2^ and 800 s/mm^2^, 800 s/mm^2^ and 1, 000 s/mm^2^ (*P* > 0.05).		

经ROC分析，4个不同b值组的ROC曲线下面积（area under curve, AUC）分别为0.831、0.876、0.813、0.785，均有诊断意义，AUC > 0.5可作为良恶性病变鉴别诊断的有效指标，且b=500 s/mm^2^时获得的ADC值的诊断效能最大（图 3），此时ADC值鉴别良恶性病变的最佳阈值为1.473×10-3 mm^2^/s，其敏感度和特异度分别为80%和84%。

**3:**
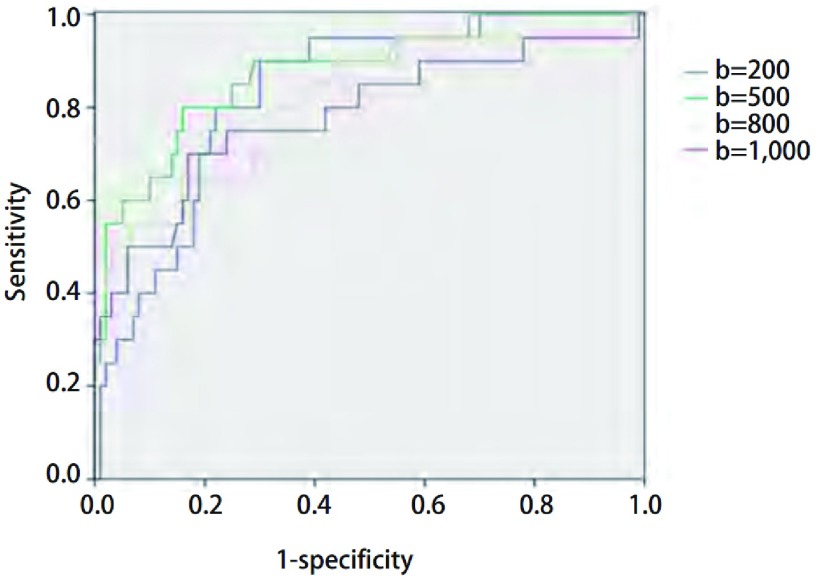
不同b值组表观扩散系数值的受试者操作特征曲线分析 Receiver operating characteristic curve (ROC) curves of apparent diffusion coefficient (ADC) value with different b values for differential diagnosis of pulmonary lesions

## 3 讨论

近年来，3.0T MR扫描仪的应用，为临床和科研提供了更高的平台，其优点是：SNR和分辨率高、图像清晰、且扫描速度更快，能够应用特殊设计的成像技术完成复杂的检查^[7, 8]^。ASSET是一种并行采集技术，利用较高的局部梯度磁场，并通过增加K空间采样位置的距离来减少K空间的采样密度，从而减少采集时间，且在小视野内通过专门重建算法，保证空间分辨力不变。ASSET技术用于EPI序列时，可缩短EPI回波链长度，减小磁场非均匀性所致的横向弛豫和质子失相位的影响，能够在一定程度上提高图像的SNR，减少磁敏感性伪影和图像变形，改善图像质量^[9]^。3.0T的超高场强和并行采集技术能够明显缩短扫描时间，减少器官和组织运动所致的伪影，易被受检者接受并取得其合作，适合胸部DWI检查。本研究在上述技术的基础上采用呼吸门控技术，患者在自由均匀呼吸下即可完成扫描，避免了由于屏气不良造成的伪影。

b值即扩散敏感因子，是在DWI检查中可由操作者选择的一个扫描参数。目前，由于MR扫描仪场强、成像序列和参数不同，胸部DWI检查尚无最佳b值可参考。b值选择应满足以下3点^[2]^：①能够清晰显示和分辨被检组织; ②能够有效抑制T2透射效应（T2 shine-through effect）对DWI的影响; ③应用尽可能高的b值以使被检组织的ADC值更接近组织真实扩散值。b值越小，DWI图像的SNR和CNR越高，但T2透射效应、灌注、宏观运动等因素对ADC值影响越大; 反之，b值越大，ADC值越接近组织的真实弥散值，但磁敏感伪影、图像几何变形等将明显降低图像SNR和CNR^[10]^。因此，b值的选择需要权衡ADC值及图像SNR和CNR两方面的得失。本研究发现，在所应用的3.0T设备和参数条件下，随b值增大，SNR逐渐下降，图像质量越差，四组b值中，CNR在b=500 s/mm^2^时最佳。先前的胸部DWI研究多在1.5T MR扫描仪上进行^[3-5, 11-13]^，推荐应用b值较高（b=500 s/mm^2^-1, 000 s/mm^2^）。本研究应用3.0T MR扫描仪所得的b值亦在此范围内，但处于低限，这可能与设备场强和主磁场均匀性差异有关。本研究结果表明应用3.0T设备，当b=500 s/mm^2^时可获得较佳的图像SNR和CNR，且能较准确反映组织扩散的真实性。

本研究还发现b=500 s/mm^2^时，AUC最大，获得的ADC值的诊断效能最大，以1.473×10-3 mm^2^/s作为诊断肺良恶性病变的界值，其敏感度和特异度分别为80%和84%，较其它三组b值时均高。应用DWI ADC值鉴别肺良恶性病变一直被很多学者所关注，但目前尚无良恶性病变的ADC值阈值标准。本研究结果与刘等^[12]^和Matoba等^[13]^应用1.5T MR扫描仪的DWI研究结果相近，他们的结果亦表明b=500 s/mm^2^时的ADC值诊断效能最大。随着3.0T及以上超高场强设备软、硬件技术的进步，胸部DWI中b值的最佳取值范围和鉴别良恶性病变的ADC值阈值标准，均还需进一步研究证实。

总之，在3.0T MR扫描仪上，采用相控阵线圈和ASSET技术DWI检查对肺内实性良恶性病变的鉴别诊断能够提供有价值的信息，DWI有望成为肺内实性良恶性病变的辅助诊断方法; 兼顾图像质量和组织扩散特性的准确程度两个方面，当b=500 s/mm^2^时获得的ADC值诊断效能较高，可作为3.0T MR DWI鉴别肺部良恶性病变的选用b值。

## References

[1] Wang J, Takashima S, Takayama F (2001). Head and neck lesions: characterization with diffusion-weighted echo-planar MR imaging. Radiology.

[2] Sun YS, Zhang XP, Tang L (2005). Diffusion-weighted MR imaging of rectal cancer: determination of b values and evaluation of displaying ability. Chin J Med Imaging Technol.

[3] Satoh S, Kitazume Y, Ohdama S (2008). Can malignant and benign pulmonary nodules be differentiated with diffusion-weighted MRI?. AJR Am J Roentgenol.

[4] Tondo F, Saponaro A, Stecco A (2011). Role of diffusion-weighted imaging in the differential diagnosis of benign and malignant lesions of the chest-mediastinum. Radiol Med.

[5] Liu H, Liu Y, Yu T (2010). Usefulness of diffusion-weighted MR imaging in the evaluation of pulmonary lesions. Eur Radiol.

[6] Zhou RC, Yu TL, Feng CC (2011). Diffusion-weighted imaging for assessment of lung cancer response to chemotherapy. Chin J Lung Cancer.

[7] Naganawa S, Kawai H, Fukatsu H (2005). Diffusion-weighted imaging of the liver: technical challenges and prospects for the future. Magn Reson Med Sci.

[8] Seo HS, Chang KH, Na DG (2008). High b-value diffusion (b=3000 s/mm^2^) MR imaging in cerebral gliomas at 3T: visual and quantitative comparisons with b=1000 s/mm^2^. AJNR Am J Neuroradiol.

[9] Willinek WA, Gieseke J, von Falkenhausen M (2003). Sensitivity encoding for fast MR imaging of the brain in patients with stroke. Radiology.

[10] Cercignani M, Horsfield MA (2001). The physical basis of diffusion-weighted MRI. J Neurol Sci.

[11] Tanaka R, Horikoshi H, Yoshida T (2011). Diffusion-weighted magnetic resonance imaging in differentiating the invasiveness of small lung adenocarcinoma. Acta Radiol.

[12] Liu HD, Yu TL, Liu Y (2010). Diffusion-weighted imaging in malignant pulmonary tumors and solid benign lesions. Int J Med Radiol.

[13] Matoba M, Tonami H, Kondou T (2007). Lung carcinoma: diffusion-weighted MR imaging-preliminary evaluation with apparent diffusion coefficient. Radiology.

